# Electrical Maxwell Demon and Szilard Engine Utilizing Johnson Noise, Measurement, Logic and Control

**DOI:** 10.1371/journal.pone.0046800

**Published:** 2012-10-15

**Authors:** Laszlo Bela Kish, Claes-Göran Granqvist

**Affiliations:** 1 Department of Electrical and Computer Engineering, Texas A&M University, College Station, Texas, United States of America; 2 Department of Engineering Sciences, The Ångström Laboratory, Uppsala University, Uppsala, Sweden; National Research & Technology Council, Argentina

## Abstract

We introduce a purely electrical version of Maxwell's demon which does not involve mechanically moving parts such as trapdoors, etc. It consists of a capacitor, resistors, amplifiers, logic circuitry and electronically controlled switches and uses thermal noise in resistors (Johnson noise) to pump heat. The only types of energy of importance in this demon are electrical energy and heat. We also demonstrate an entirely electrical version of Szilard's engine, i.e., an information-controlled device that can produce work by employing thermal fluctuations. The only moving part is a piston that executes work, and the engine has purely electronic controls and it is free of the major weakness of the original Szilard engine in not requiring removal and repositioning the piston at the end of the cycle. For both devices, the energy dissipation in the memory and other binary informatics components are insignificant compared to the exponentially large energy dissipation in the analog part responsible for creating new information by measurement and decision. This result contradicts the view that the energy dissipation in the memory during erasure is the most essential dissipation process in a demon. Nevertheless the dissipation in the memory and information processing parts is sufficient to secure the Second Law of Thermodynamics.

## Introduction

Heat engines [1] utilize temperature differences to produce work while “heat demons” [2]–[8] employ information about instantaneous amplitudes of thermal fluctuations and execute control to produce a temperature difference and/or work. There has been an upsurge of interest in “heat demons”, as evidenced from an extensive recent literature [9]–[18]. Furthermore there is an old debate [8]–[14], [19]–[21] on the question whether the energy dissipation due to erasure of information in the memory is the fundamental process to save the Second Law of Thermodynamics or if the generation of new information via measurement and decision [2], [13] and control requirements [9] is more important.

This paper introduces new and improved types of demons: they are purely electrical systems which employ the thermal voltage noise (Johnson noise) of resistors in thermal equilibrium. One of the demons pumps heat (similarly to Maxwell's demon) and the other produces work (similarly to Szilard's engine). Their common characteristics is that nothing but electrical energies appear in the fluctuations, measurement, decision, control and memory parts of the demons, and that all of the energy loss is due to dissipative electrical transport. This situation is very different from that of earlier versions of Maxwell's demon and Szilard's engine wherein various forms of energies are (implicitly) employed in the various components. In the case of Maxwell's demon, for example, the detection of an approaching molecule and its velocity require photoelectronics-based sensing that includes laser light (photon energy), photodiodes, electrical signals and energy, and an electromechanical system to drive the trapdoor, as well as related kinetic and potential mechanical energies. Our demon, which is a linear system, is also different in nature from ratchet-based demons [5] and Brillouin's diode-based ones [6], [7] because those utilize nonlinearity instead of measurement, decision and control. Similarly, in Szilard's engine, detecting the location of the molecule, controlling the lever and repositioning the piston require photonic, electrical and mechanical energies and imply related forms of energy dissipation.

Complete evaluations of the various energy dissipation channels have not been performed in earlier work on Maxwell's demon and Szilard's engine, which may be associated with difficulties to assess the transfer of energies between their different forms. However our purely electrical demons, which are introduced in this paper, offer fundamental advantages and provide a convenient methodology for such analyses.

## Discussion and Results

### 1. Electrical Maxwell demon utilizing Johnson noise and control

#### 1.1 General description

**Figure 1:**
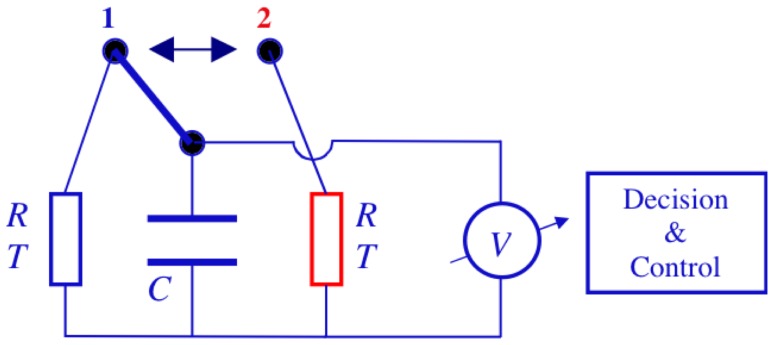
Electrical Maxwell demon with Johnson noise in stage 1. The temperature *T* is the same in both resistors. The thick line indicates the moving part of the switch and the double-ended arrow the switching positions.

The electrical Maxwell demon described below employs the Johnson noise of resistors as well as measurement, decision, logic operations and control for pumping heat. One should note that control systems in general contain units for making measurements, decision and executing control. Figure 1 shows an electrical Maxwell demon in its starting stage 1 extracting energy from the resistor to the left. The switch is in position 1, and the Johnson noise current of the resistor yields voltage fluctuations 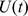 in the capacitor with variance
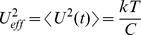
(1)in accordance with Boltzmann's energy equipartition theorem. It leads to a mean energy of 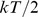 in the capacitor [1], where 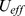 is the effective noise voltage (root mean square, RMS, voltage) on the capacitor 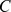. This voltage fluctuation is a Gaussian process with exponential relaxation and correlation time (relaxation time) 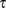 expressed as
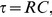
(2)where 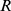 is the resistance, and with an amplitude distribution function 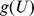 given by
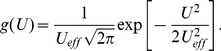
(3)


**Figure 2:**
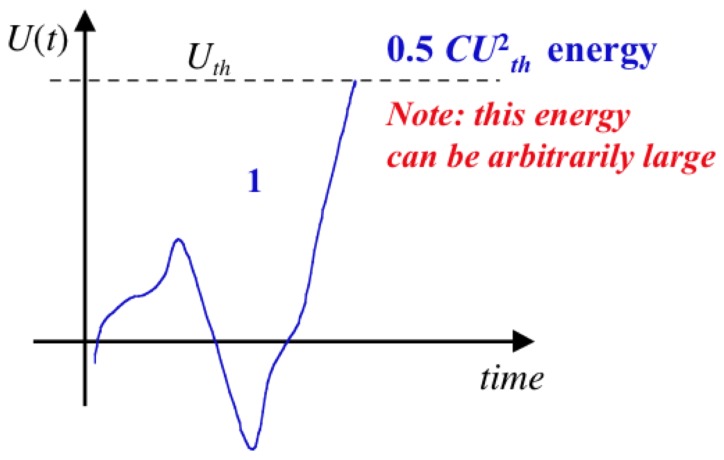
Voltage fluctuations in the capacitor during stage 1 for an electrical Maxwell demon with Johnson noise. (Note that the real voltage time function typically exhibits more random fluctuation events, as discussed later.)

**Figure 3:**
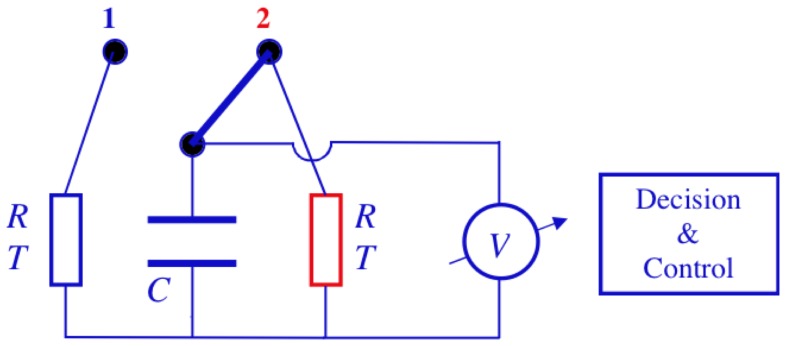
Electrical Maxwell demon with Johnson noise in stage 2.

**Figure 4:**
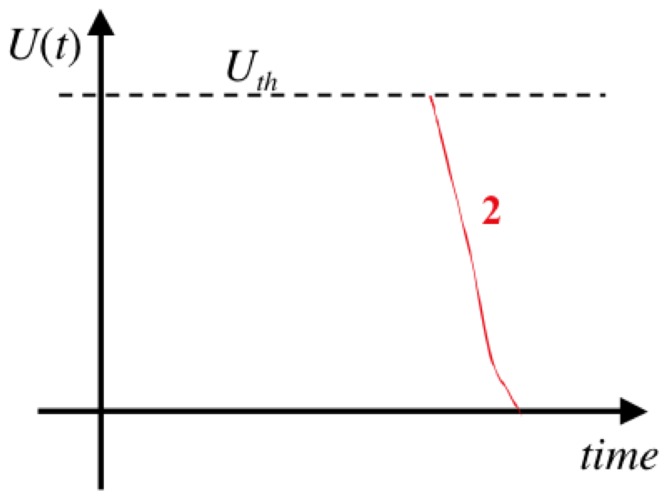
Voltage fluctuations on the capacitor during stage 2 for an electrical Maxwell demon with Johnson noise. (Note that real voltage fluctuations typically exhibit more random events, as discussed later.)

The voltage 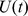 is monitored as illustrated in Figure 2, and the demon stays in stage 1 until the voltage reaches a chosen arbitrary threshold value 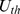, which corresponds to the energy
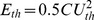
(4)on the capacitor. The switch is then flipped to position 2, see Figure 3, and the demon gets into its stage 2 wherein the capacitor is discharged and the energy 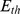 is pumped into the resistor on the right. In stage 2, 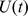 is monitored on the capacitor, as shown in Figure 4, and when it reaches the zero threshold level with 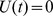 the switch is flipped into its position 1 shown in Figure 1. Thus the demon gets into stage 1 again, and the full cycle of the demon is completed through this step.

**Figure 5:**
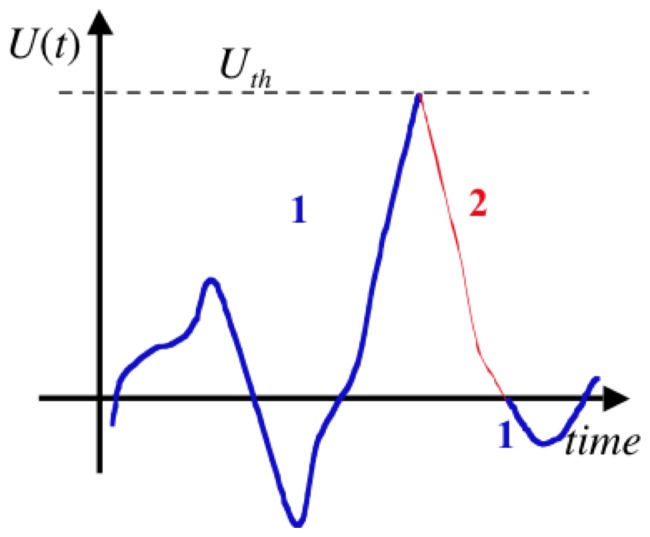
Voltage fluctuations in the capacitor, indicating the role of the two threshold levels *U_th_* and 0 during the whole cycle, and the beginning of a new cycle.

By the end of stage 1, the capacitor has extracted the energy 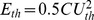 from the resistor on the left, and this energy is fully dissipated in the resistor on the right by the end of stage 2. When the demon gets back to stage 1 to start a new cycle, the energy in the capacitor is zero. Thus by the end of the new stage 1 process, the capacitor again extracts 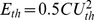 from the resistor on the left, and this energy will once more be fully dissipated in the resistor on the right by the end of the new stage 2. Figure 5 illustrates voltage fluctuations during the whole cycle.

It is important to note that, in an ideal system, the heat pumped during a single cycle does not have an upper limit because, according to Eq. 2, the threshold amplitude 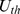 (and thus the energy 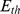) can be chosen to be arbitrarily large.

#### 1.2. Cycle duration versus extracted heat/work during a cycle

The cycle frequency of Maxwell's demon is usually not part of its energy balance, but we note that an increase of the threshold 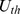 will cause an exponential slowdown of the demon as a result of the increased cycle time needed to reach 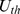, as further discussed later. The duration of the exponential discharge process in stage 2 scales logarithmically with the pumped energy (threshold energy) 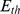, whereas the duration of stage 1 scales exponentially in the limit of large 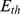, as discussed below. Thus the main question is the average duration of stage 1, i.e., the mean first-passage time 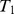 of the voltage fluctuation of the capacitor between zero and 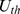 when this energy is much greater than the average thermal energy in the capacitor; the threshold is given by
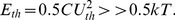
(5)


This expectation agrees with an earlier, analytic treatment of Gaussian noise with exponential relaxation, wherein it was derived in an asymptotic limit [22]. Note that first passage times belong mostly to the field of “unsolved problems of noise”, and analytic results (e.g. [22]–[24]) exist only for the asymptotic limit indicated by Eq. 5. In the present analysis we use results from the analytic treatment in [22].

A dimensionless first passage time 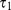 from zero amplitude to the level 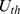 is obtained [22] according to
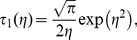
(6)where 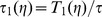. Here 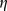 is the dimensionless threshold 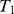 and 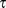 is the relaxation time of the Lorentzian. In our system the dimensionless threshold is 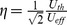, where 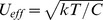 is the effective value of the noise. Thus the real first passage time 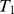 from zero amplitude to level 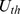 is
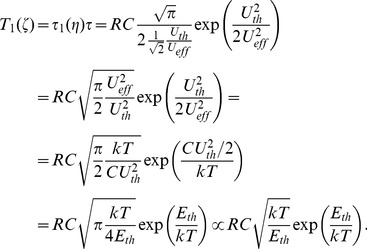
(7)


In conclusion, even though an arbitrarily large energy 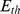 can be transferred by the Maxwell demon type heat pump within a cycle, the cycle duration scales exponentially with this energy, which means that increasing 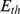 results in an exponential scaling down of the mean power.

#### 1.3. On energy requirement and dissipation

**Figure 6:**
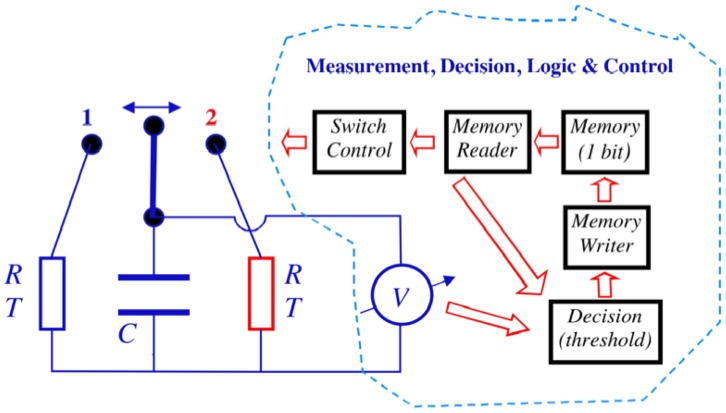
Electrical Maxwell demon showing a block scheme of the building elements for measurement, decision and control. The voltmeter and the threshold device create new information, and the rest of the system only processes or utilizes this binary information.

**Figure 7:**
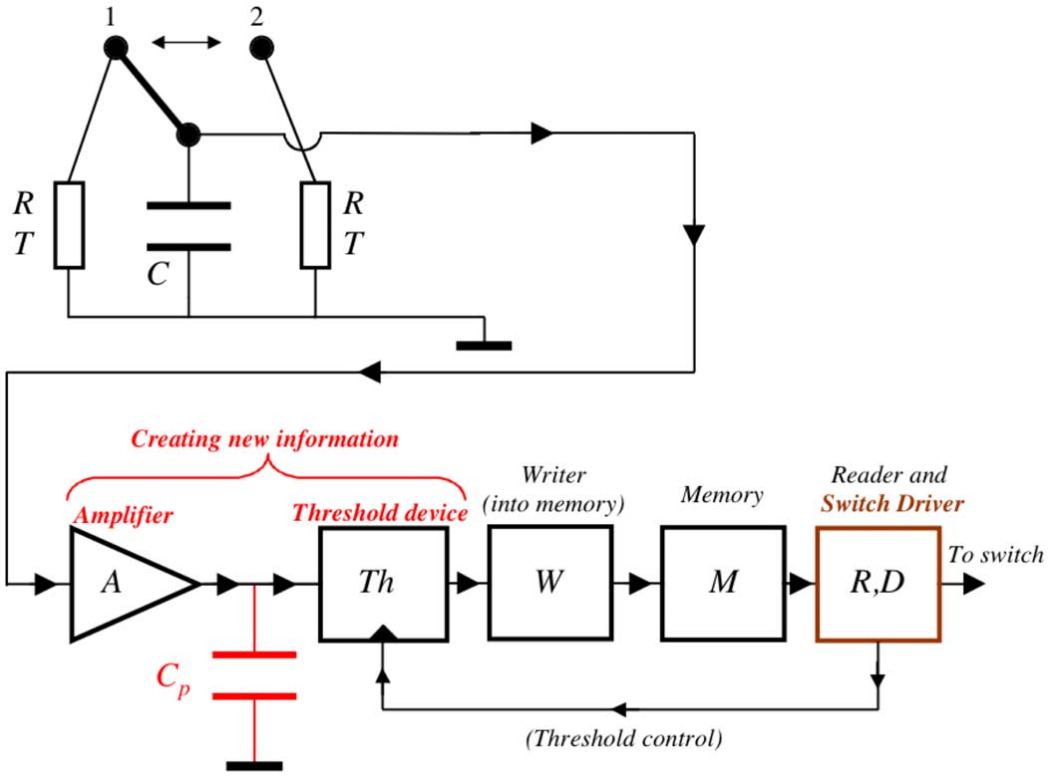
Possible realization of an electrical Maxwell demon. Some of the elements can be joined in other realizations, but the energy dissipation sources described here remain.

In this section we discuss the energy dissipation in the limit of high threshold, 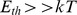 (where Eq. 7 holds), and small error probability. A full assessment of the energy dissipation needed to run the demon requires consideration of all of its building elements. Such analyses have not been common in prior work because the demon's functional characteristics have usually not been described in sufficient detail. To avoid mistakes caused by such deficiencies, Figure 6 shows the complete functional block scheme required to realize Maxwell's demon. A possible physical realization of this scheme is given in Figure 7.

It is important to identify the dominant causes of energy dissipation. The first two stages of the measurement, decision, logic and control unit in Figure 6 are the amplifier and the threshold device in Fig. 7, which represent the measurement and decision. For example, the output of the threshold device can be a positive or negative pulse representing the moment when the switch state must be changed from 1 to 2 or from 2 to 1 and triggering the memory to alter its state accordingly. The memory stores binary, single-bit information—a high or low value—and these two-bit states signify the actual state (1 or 2) of the switch. During a single cycle of the demon, the single-bit information cycles through its two states low-high-low or high-low-high depending on the choice of the designer of the system: whether the low-bit represents the switch-state 1 and the high-bit the switch-state-2, or vice versa.

The reader and switch driver device is basically an amplifier system that reads out the memory status and checks the voltage controlled switches, which either make a connection between the capacitor and point 1 while breaking the former connection to point 2, or vice versa. Thus these are two electronically controlled switches—between the capacitor and 1 and between the capacitor and 2, respectively—driven in alternate fashion.

We now consider the elements for handling the binary information and note that according to Brillouin's negentropy principle [6], [7] the processing of each bit of information will dissipate energy of at least 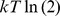, and processing with acceptable error probability 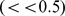 will require even greater energy dissipation 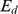 [9], [25]. Within the correlation time (reciprocal bandwidth) of thermal fluctuations and in the small-error probability limit [9], [25] the lower boundary of energy dissipation can be described by the Rice formula [25]. The required energy is logarithmically divergent when the error probability goes to zero according to
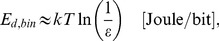
(8)where 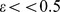 is the bit error probability during the correlation time 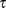. Demanding the same numerical value of the error probability [9] for a longer cycle time (or clock period) 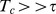 makes the energy dissipation increase logarithmically as a result of the additivity of independent errors generated in different non-overlapping correlation time intervals. It follows that
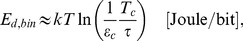
(9)where 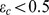 is the bit error probability during 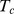. Using the result of Eq. 7 we obtain the following relation for the lowest possible energy dissipation of each binary switching element (transistor, etc) in the demon:
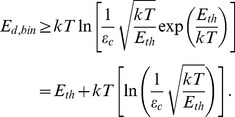
(10)


We conclude that the Second Law of Thermodynamics is satisfied by even a single binary unit (such as any logic gate or the memory) for the case of a fixed 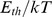 ratio and error probability approaching zero. The energy dissipation scales roughly with the energy threshold 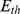, i.e., in the small error limit it scales with the pumped energy.

Turning now to the major sources of energy dissipation, we consider the amplifier and the threshold device, which are the analog circuitry elements where new information is created. Their energy dissipation scale exponentially with *E_th_* and is much larger than the amount required by the Second Law of Thermodynamics. Theoretically, if the chosen threshold level 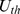 is large enough, the amplifier is not needed and it is sufficient with a threshold device. However, any such device can be modeled as an amplifier with either saturation and/or positive feedback, and thus we keep the amplifier stage because it helps illustrate the point where the energy dissipation scales exponentially with 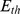.

In order to elucidate the origin of the amplifier's energy dissipation it should be realized that any amplifier has a component of inertia. To choose a simple illustrative example, a hydraulic amplifier has inertia due to the mass of its components. In the present case of an electrical voltage amplifier, inertia ensues from parasitic capacitances and inductances. We demonstrate this issue of electrical inertia by considering the final capacitor, positioned at the point where the threshold device makes the decision about the threshold crossing, denoted *C_p_* in Figure 7. For the sake of simplicity we assume that the amplification is unity in the amplifier. Under thermal equilibrium, thermal voltage fluctuations (Johnson noise) appear on the capacitor with an RMS value 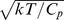, which corresponds to a mean thermal energy of *kT*/2 in the capacitor. If this Johnson noise is not actively damped it must be kept at a much lower level than the main Johnson noise of the demon we are monitoring, i.e., 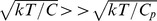. This means that 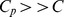 in order to reduce erroneous threshold crossings caused by the parasitic noise. We note in passing that it can be shown that active damping of the parasitic Johnson noise would cause greater energy dissipation than the value discussed below [26].

**Figure 8:**
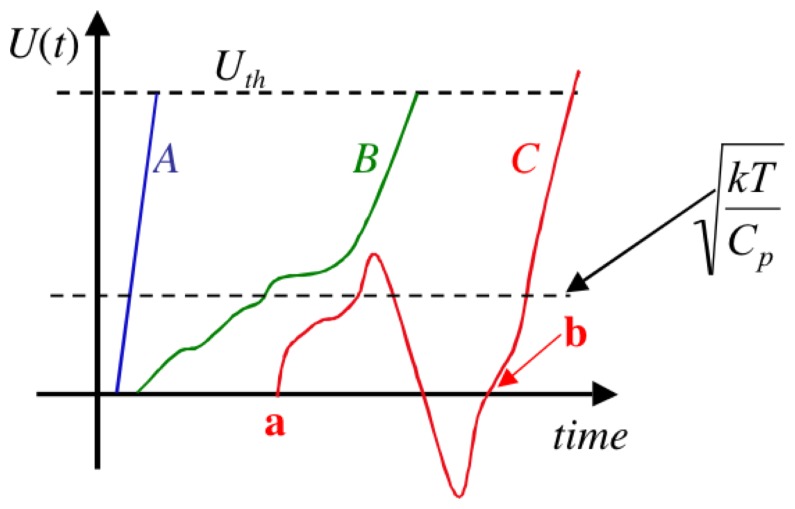
Illustration of the main source of energy dissipation in the electrical Maxwell demon. An exponential number of random fluctuation events as a function of the threshold energy take place before reaching the threshold. The energy dissipation between the points **a** and **b** represent complete energy loss and do not contribute to the process of attaining the threshold.

Figure 8 illustrates three possible trajectories of the voltage within stage 1 as its amplitude goes from zero to 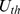. Curves *A* and *B* represent monotonically increasing functions which reach 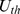 with relatively modest energy investment, i.e., 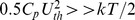 plus the thermalization losses due to the resistances, etc (not shown in the circuitry). However curve *C* is qualitatively different and non-monotonic. This feature causes serious additional energy dissipation and all of the energy invested in the trajectory from points a to b is completely lost. For example, in the case of a symmetric power supply (i.e., with electrodes: +, −, and zero (ground)) the parts of the trajectory with positive velocity (increasing amplitude) are supplied by the positive electrode of the power source, while the decreasing parts of the trajectory are supplied by the negative electrode, when in both cases the current flows into the ground. Both of these currents represent positive power extracted from the positive and negative parts of the supply, respectively. Even though a net energy was dissipated in the voltage-path from a to b, the whole charging process is back to zero at b, and all of the earlier invested energy was lost.

How many such dissipative random fluctuation events will the voltage on the capacitors exhibit, and how large are these fluctuations? During the correlation time 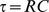 of the noise in the main capacitor the noise typically goes through at least one large wave ranging though the +/− RMS amplitude interval. This means that the related energy dissipation 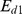 during the correlation time of the main noise is of the order
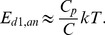
(11)


According to Eq. 7 the expected number of correlation time (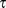) periods during the dominant stage 1 part of the clock cycle is 
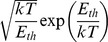
 so that the total energy dissipation is of the order
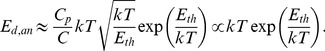
(12)


Thus the dominant energy dissipation during a single cycle in the large-threshold energy limit is
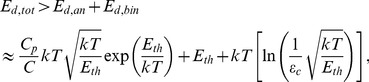
(13)and hence the approximate scaling in this limit is
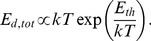
(14)


In conclusion, the dominant energy dissipation in the electrical Maxwell demon takes place in the part which generates the new information and is due its analog processing yielding an exponential dependence of the number of random voltage oscillations during a single cycle.

### 2. Electrical Szilard engine utilizing Johnson noise and control

The principles of the Johnson noise driven electrical Szilard engine are the same as those of the electrical Maxwell demon though heat is not pumped but work is executed by a moving capacitor plate used as a piston [1], [27]. The control and all other considerations are inherently the same as for Maxwell's demon except the fact that the Szilard engine requires a twice as large system as the Maxwell demon. Thus two single-bit memories are needed and the related voltage monitoring and switch control must be performed over two capacitors.

**Figure 9:**
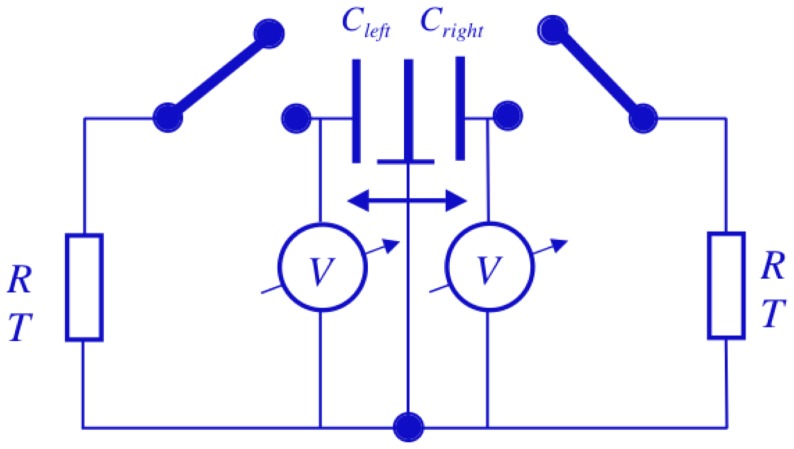
Electrical Szilard engine extracting heat from the resistor and executing work on the piston, i.e., the movable capacitor plate.

Figure 9 shows the main elements and circuitry of the electrical Szilard engine except the decision, logic and control components which are identical to the corresponding parts in Maxwell's demon. The electrical Szilard engine extracts heat from the resistor and executes work on the piston, which is the moving joint capacitor plate of the two capacitors at the middle.

The left capacitor has its maximum capacitance 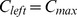 when the piston is at the left end while the right capacitor then attains its minimum capacitance 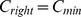. In the opposite case, when the piston is at the right end, the left capacitor reaches its minimum value 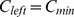 while the right capacitor has its maximum capacitance 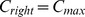. The capacitor with the minimum capacitance will be charged up to the work charge 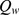 at different points of the cycle; they correspond to a threshold voltage 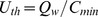 as shown in Figure 2. The corresponding electrical charging energy—analogously to the case of Maxwell's demon—is much greater than the thermal equilibrium value, i.e.,
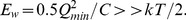
(15)


**Figure 10:**
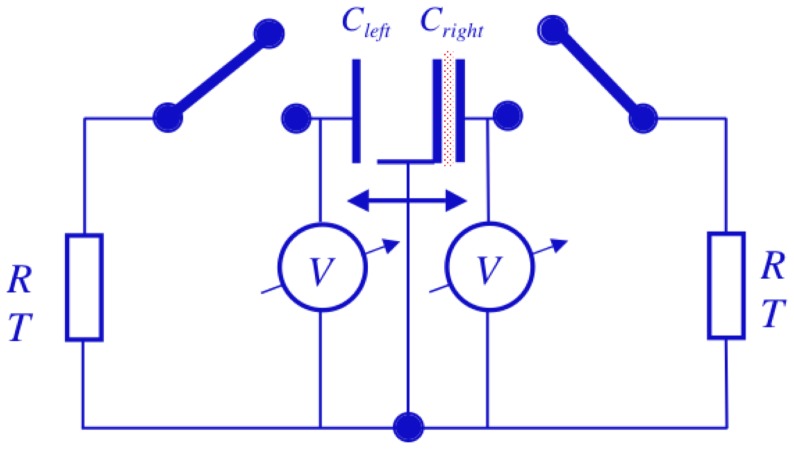
Arbitrarily chosen starting point of the cycle for the electrical Szilard engine. The piston is at the right end and the capacitor at the right has the work charge *Q_w_*. The corresponding electrical energy is *E_w_*≫*kT*/2.

Figure 10A shows an arbitrarily chosen starting point of the cycle. The piston is at the right end and the capacitor to the right has the work charge 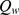. The voltage on the right capacitor is 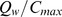. The energy difference, compared to the situation with the opposite position of the piston at the same charge, is
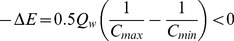
(16)implying that the piston has a negative potential energy and sits in a potential well. We suppose that
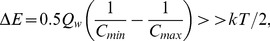
(17)i.e., the piston remains at the bottom of this potential barrier, which is in the vicinity of the right end, while it executes its mechanical Brownian motion.

**Figure 11:**
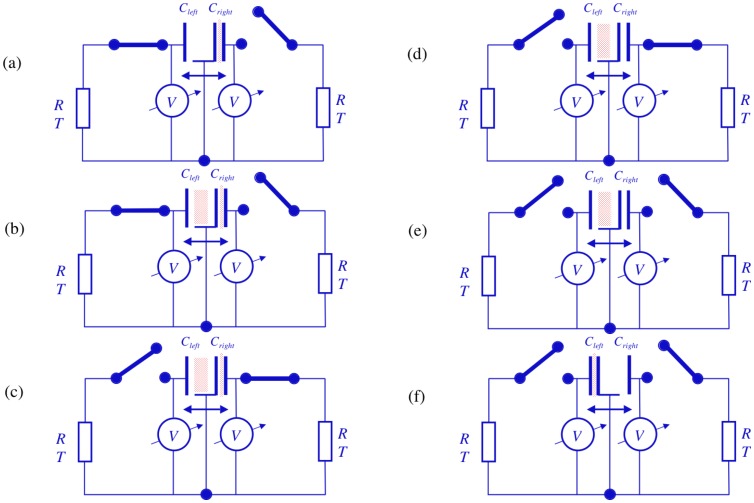
Operation of the first half cycle for the electrical Szilard engine. (a): The left switch is closed to charge the left capacitor. (b): Johnson noise current yields charge fluctuations in the left capacitor, i.e., a Gaussian noise in the charge. (c): As soon as the left capacitor reaches *Q_w_*, the left switch is abruptly opened to keep the charge in the left capacitor and the right switch is closed to discharge the capacitor on the right. (d): The charge in the right capacitor decays exponentially and, within a time interval of a few *RC*, thermalizes and crosses the zero charge (zero voltage) level. (e): The right switch is abruptly opened and the zero charge (zero voltage) state is preserved in the right capacitor. (f): The force due to the working charge in the left capacitor moves the piston to the left end, which is the end of the half-cycle; the piston is at the left end and the capacitor at the left has the work charge *Q_w_*.

Figure 11 illustrates the operation of the first half-cycle for the electrical Szilard engine. In Figure 11A the left switch is closed in order to charge the left capacitor and the Johnson noise current of the left resistor yields charge fluctuations in the left capacitor, which is a Gaussian noise of the charge; see Figure 10B. After an exponentially long waiting time characterized by Eq. 7 the left capacitor, which originally had zero charge, is charged up to the working charge 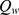 and energy 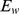 given by Eq. 15. At that moment the left switch is abruptly opened to keep the charge in the left capacitor and the right switch is closed to discharge the capacitor on the right as shown in Figure 10C. The charge in the right capacitor decays exponentially and, within a time interval of a few *RC*, thermalizes and crosses the zero charge (zero voltage) level; see Figure 10D. At that moment, as illustrated in Figure 10E, the right switch is abruptly opened and the zero charge (zero voltage) state is preserved in the right capacitor. Then the electrostatic force due to the working charge in the left capacitor moves the piston to the left end, as shown in Figure 10F, while, in accordance with the energy conservation law, the piston executes positive work amounting to 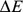. This is the end of the half-cycle. The piston is at the left end and the capacitor at the left has the work charge 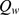.

**Figure 12:**
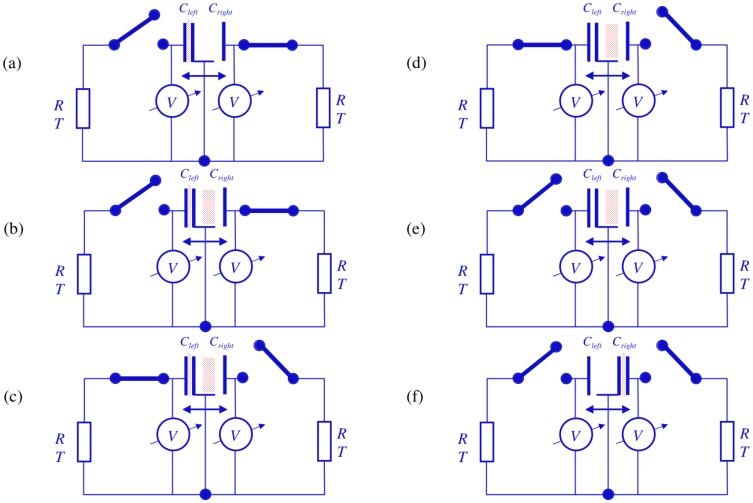
Operation of the second half cycle for the electrical Szilard engine. (a): The right switch is closed to charge the right capacitor. (b): Johnson noise current yields charge fluctuations in the right capacitor, i.e., a Gaussian noise in the charge. (c): As soon as the right capacitor reaches *Q_w_* the right switch is abruptly opened to keep the charge in the right capacitor and the left switch is closed to discharge the capacitor on the right. (d): The charge in the left capacitor decays exponentially and, within a time interval of a few *RC*, thermalizes and crosses the zero charge (zero voltage) level. (e): The left switch is abruptly opened and the zero charge (zero voltage) state is preserved in the left capacitor. (f): The force due to the working charge in the right capacitor moves the piston to the right end, which is the end of the full cycle and the same state as where the analysis started; see Figure 10. The piston is at the right end and the capacitor in the right has the work charge *Q_w_*.

The remainder of the full cycle comprises the same processes as during the first half with the proper change of the indices signifying “left” and “right” of capacitors and switches, as elucidated in Figure 12.

Thus the full cycle has been carried out with purely electronic control based on the information from the measurement of charge or voltage in the capacitors. It should be noted that the system is free from the major deficiency of the original Szilard engine, which is that its piston must be artificially relocated into the initial position at the end of the cycle. The two switches represent four possible states and two bits of information, and consequently the unit for measurement, decision, logic and control is basically twice that of the system shown for the electrical Maxwell demon, while the energy dissipation essentially doubles. Thus the energy dissipation in the binary part, including in the memory, is negligible compared to that in the analog part that executes the measurement and creates the new information.

## Methods and Conclusions

Two electrical demons utilizing Johnson noise, measurement, decision, logic and control were introduced; they are new versions of Maxwell's demon and Szilard's engine. We showed all of the necessary building elements and analyzed the cycle time versus the energy output. Both demons have an arbitrarily large energy output during a single cycle, and the cycle duration and energy dissipation scale exponentially with this energy output. The exponentially scaling energy dissipation occurs in the analog parts of the demons, where the creation of new information (measurement and decision) takes place. In the binary part, including the memory, the energy dissipation scales only linearly with the energy output and even this dissipation is enough to satisfy the Second Law of Thermodynamics due to the enhanced energy threshold requirements caused by the exponentially long cycle duration and the requirement of keeping the error probability small.
